# A common neural substrate for processing scenes and egomotion-compatible visual motion

**DOI:** 10.1007/s00429-020-02112-8

**Published:** 2020-07-09

**Authors:** Valentina Sulpizio, Gaspare Galati, Patrizia Fattori, Claudio Galletti, Sabrina Pitzalis

**Affiliations:** 1 Department of Biomedical and Neuromotor Sciences-DIBINEM, University of Bologna, Piazza di Porta San Donato 2, 40126 Bologna, Italy; 2 Department of Cognitive and Motor Rehabilitation and Neuroimaging, Santa Lucia Foundation (IRCCS Fondazione Santa Lucia), Rome, Italy; 3 Brain Imaging Laboratory, Department of Psychology, Sapienza University, Rome, Italy; 4 Department of Movement, Human and Health Sciences, University of Rome ‘‘Foro Italico’’, Rome, Italy

**Keywords:** Optic flow, Scene perception, Functional magnetic resonance, Brain mapping, OPA, V3A

## Abstract

Neuroimaging studies have revealed two separate classes of category-selective regions specialized in optic flow (egomotion-compatible) processing and in scene/place perception. Despite the importance of both optic flow and scene/place recognition to estimate changes in position and orientation within the environment during self-motion, the possible functional link between egomotion- and scene-selective regions has not yet been established. Here we reanalyzed functional magnetic resonance images from a large sample of participants performing two well-known “localizer” fMRI experiments, consisting in passive viewing of navigationally relevant stimuli such as buildings and places (scene/place stimulus) and coherently moving fields of dots simulating the visual stimulation during self-motion (flow fields). After interrogating the egomotion-selective areas with respect to the scene/place stimulus and the scene-selective areas with respect to flow fields, we found that the egomotion-selective areas V6+ and pIPS/V3A responded bilaterally more to scenes/places compared to faces, and all the scene-selective areas (parahippocampal place area or PPA, retrosplenial complex or RSC, and occipital place area or OPA) responded more to egomotion-compatible optic flow compared to random motion. The conjunction analysis between scene/place and flow field stimuli revealed that the most important focus of common activation was found in the dorsolateral parieto-occipital cortex, spanning the scene-selective OPA and the egomotion-selective pIPS/V3A. Individual inspection of the relative locations of these two regions revealed a partial overlap and a similar response profile to an independent low-level visual motion stimulus, suggesting that OPA and pIPS/V3A may be part of a unique motion-selective complex specialized in encoding both egomotion- and scene-relevant information, likely for the control of navigation in a structured environment.

**Electronic supplementary material:**

The online version of this article (10.1007/s00429-020-02112-8) contains supplementary material, which is available to authorized users.

## Introduction

Optic flow is an important cue for monitoring our movements in the surrounding environment. Beyond its relevance for self-motion perception, perception of egocentric flow motion might also be crucial for scene recognition, since scene views change dynamically due to self-motion.

Traditionally, two distinct neural substrates are considered for optic flow processing and scene recognition: a dorsal ‘action’ pathway that mediates the on-line processing of visual motion information, likely aimed at monitoring self-to-object spatial relationships to guide goal-directed actions in dynamic visual environments, and a ventral ‘perception’ pathway that mediates the analysis of visual attributes of the visual world to allow object and scene recognition (Goodale and Milner 1992). Up to now, how visual information carried out in these two separate systems are subsequently integrated into a unified visual percept remains a matter of debate.

Optic flow processing simulating self-motion (egomotion; Gibson 1950) has been ascribed to a network of dorsal higher-level visual and multisensory cortical regions (egomotion-selective regions) including the medial parieto-occipital areas V6 and V6Av (V6 complex or V6+; Pitzalis et al. 2006, 2010, 2013b; Cardin and Smith 2010), the posterior segment of the intraparietal sulcus (pIPS), a location remarkably coincident with the dorsal part of retinotopic area V3A (Tootell et al. 1997; Pitzalis et al. 2010), the cingulate sulcus visual areas (CSv and pCi; Wall and Smith 2008; Serra et al. 2019), the posterior insular cortex (PIC; Frank et al. 2014), and a parietal motion region located in a region similar to that of the putative human area VIP (IPSmot, Pitzalis et al. 2013c; Bremmer et al. 2001; Sereno and Huang 2006; Cardin and Smith 2010). Despite their general involvement in processing optic flow or egomotion-compatible visual stimuli (Cardin and Smith 2010), these motion-sensitive areas are likely differently recruited in guiding egomotion. For instance, in a recent paper (Serra et al. 2019), we showed that while the most posterior areas V6+, pIPS/V3A, and IPSmot/VIP are involved in the visual analysis of scenes (likely to help the interaction with the surrounding objects while moving through a complex environment; see Galletti and Fattori 2018), the most anterior areas pCi, PIC and CSv are mainly implicated in motor control during locomotion (see Smith et al. 2018 for a similar interpretation of the CSv role), being activated by a motor task requiring long-range leg movements. Although less consistently, responses to optic flow have been also found in the lateral occipitotemporal MT complex (MT+; Cardin and Smith 2010; Serra et al. 2019) and in the dorsal margin of the postcentral sulcus, in a portion of cortex likely corresponding to the human homolog of the macaque vestibular area 2v (putative 2v or p2v; Guldin and Grüsser 1998; Cardin and Smith 2010), i.e., a multisensory area, containing neurons that respond to both vestibular and optokinetic stimulation (Buttner and Buettner 1978).

Scene recognition, which has been extensively studied by neuroimaging in humans (see Epstein 2008; Julian et al. 2018 for reviews), has been ascribed to several ventromedial posterior cortical regions (scene-selective regions), such as the parahippocampal place area (PPA), the retrosplenial complex (RSC), and the occipital place area (OPA). Scene-selective areas are also involved in different processes. Beside their general involvement in representing navigationally relevant visual stimuli such as scenes and buildings (Epstein et al. 1999; Epstein and Higgins 2007; see also Epstein 2008), they have complementary functions, with the PPA more concerned with representation of the local visual scene and discrimination of different views (Park and Chun 2009; Sulpizio et al. 2013, 2014, 2016b), and RSC more concerned with real and imagined navigation (Ino et al. 2007; Wolbers and Büchel 2005), visuo-spatial mental imagery of familiar environments (Boccia et al. 2015), retrieval of environment-centered information (Committeri et al. 2004; Galati et al. 2010; Sulpizio et al. 2013, 2016b), and encoding of permanent landmarks (Auger et al. 2012; Auger and Maguire 2013). More recently, a few studies have disclosed the role of OPA in spatial cognition, showing that it encodes environmental boundaries (Julian et al. 2016) and local navigational affordances (Bonner and Epstein 2017) and represents first-perspective motion information in the immediately visible scene (Kamps et al. 2016). In few words, while PPA and RSC are implicated in identification of places/contexts and in supporting spatial transformations necessary for reorientation, respectively, OPA contains information about where a navigator can move in the scene (see Julian et al. 2018 for a review).

Only recently, a growing number of studies have explored the functional link between brain areas processing visual egomotion and scene perception. For example, Korkmaz Hacialihafiz and Bartels (2015) found that scene-selective regions were modulated by visual motion. In particular, PPA showed a significant interaction between scene content (scene vs. scrambled) and motion (motion vs. static), with similar trends observed in RSC and OPA. Schindler and Bartels (2016), using a fully controlled virtual paradigm mimicking lateral self-motion in front of a depth-layered three-dimensional scene, found parallax specific responses in the transverse occipital sulcus (OPA); they also observed that this region showed increased functional connectivity with PPA during motion parallax as compared to a low-level control condition. Additionally, an optic flow-dependent modulation of functional connectivity has been found between the early visual cortex and both visual egomotion- and scene-selective areas (Schindler and Bartels 2017).

Taken together, these studies provide direct evidence of a functional interplay between dorsal egomotion-selective and ventral scene-selective regions. However, an open issue is whether visual motion and scene perception are computed in isolation within egomotion- and scene-selective regions, respectively, or whether they are set through reciprocal interactions between these regions. Here, we hypothesized that dorsal egomotion- and ventral scene-selective regions might cooperate during processing of visual cues relevant for navigation, possibly to facilitate spatial updating and scene reconstruction within the three-dimensional environment. This possible link would suggest that egomotion and scene perception are not processed independently. To test this hypothesis, we probed the sensitivity of these two groups of regions to the non-preferred stimulus category. We, thus, used two well-known “localizer” fMRI experiments, consisting in passive viewing of navigationally relevant stationary stimuli such as buildings and places (scene/place stimulus; Sulpizio et al. 2013, 2014, 2016a, 2018) and coherently moving fields of dots (high-level visual motion stimulus or flow fields, Pitzalis et al. 2010) to interrogate egomotion-selective regions (V6+, pIPS/V3A, IPSmot/VIP, CSv, pCi, PIC) and scene-selective regions (PPA, RSC, OPA), respectively. Results showed the existence of a common neural substrate for processing scene and egomotion-compatible optic flow, since the two caudal-most egomotion-selective regions (V6+ and pIPS/V3A) also preferentially responded more to navigational scenes compared to faces (although at different extent), and all the scene-selective regions (PPA, RSC, OPA) also responded more to egomotion-compatible optic flow compared to random motion.

Since OPA and pIPS/V3A showed a similarity in brain position and functional response to an independent low-level visual motion stimulus, we further investigated the degree of overlap between these two regions on the cortical surface reconstruction of each individual hemisphere to check whether they actually belong to different or the same cortical areas. Our results suggest that OPA and pIPS/V3A are part of a unique motion-selective complex.

## Methods

### Participants

We reanalyzed data of one hundred thirty-four healthy adults (64 females, mean age 27.2, SD 4.7) who participated to previous studies from our lab (Boccia et al. 2015, 2017a, b, 2019; Pitzalis et al. 2010, 2013c, 2019; Tosoni et al. 2015; Tullo et al. 2018). 61 subjects (32 females, mean age 28.2, SD 4.1) were administered a scene/place stimulus, 96 subjects (50 females, mean age 27.2, SD 4.9) were administered a visual motion stimulus (flow fields) and 23 subjects both (15 females, mean age 29.3, SD 3.7). A sub-sample of 35 subjects (18 females, mean age 27.9, SD 3.9) was also administered an additional low-level visual motion stimulus (low-contrast radial motion or radial rings), the stimulus originally used to functionally map the human middle temporal area (Tootell et al. 1995). All participants were right-handed, as assessed by the Edinburgh Handedness Inventory (Oldfield 1971) and had normal or corrected-to-normal vision. All volunteers had given their written informed consent to participate, and the original studies had been approved by the research ethics committees either at Fondazione Santa Lucia in Rome or at University G D’Annunzio in Chieti, according to the Declaration of Helsinki.

### Experimental paradigm

Participants completed two main fMRI experiments, consisting in passive observation of scenes/places and of flow fields, respectively.

**Fig. 1:**
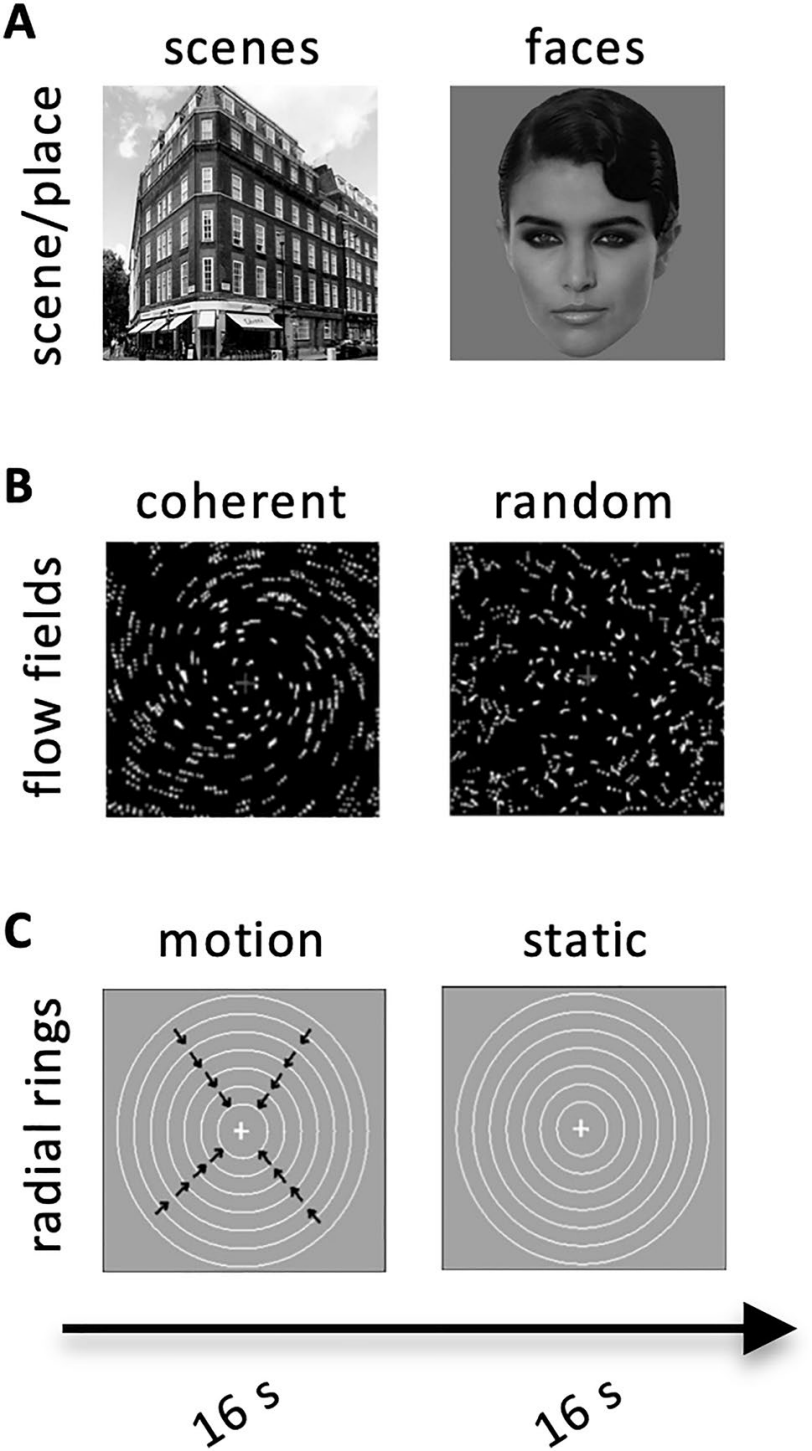
Experimental paradigms. Schematic representation of the stimuli used to localize and test scene- and egomotion-selective regions. **a** Scene/place stimulus: 16-s blocks of pictures of places (indoor and outdoor) were interleaved with 16-s blocks of human faces (male and female) and with 15-s block of fixation; **b** High-level visual motion stimulus (also called flow fields): 16-s blocks of coherently moving fields were interleaved with 16-s blocks of randomly moving fields. **c** Low-level visual motion stimulus (also called radial rings): 16-s blocks of radial motion (outward and inward) were interleaved with 16-s blocks of stationary rings

Figure 1a shows examples of scene/place stimuli, inspired from the original paradigm by Epstein and Kanwisher (1998) and described in detail in Sulpizio et al. (2013). Briefly, participants were instructed to maintain central fixation while presented with eight 16-s blocks of 240 × 240-pixel gray-scale digitized photographs of faces and scenes/places presented for 300 ms every 500 ms, interleaved with 15-s fixation blocks periods. Photos of places consisted of common indoor (50%) and outdoor (50%) scenes. Photos of faces represented faces with neutral expressions of male (50%) and female (50%) young adults. Faces were set on a solid gray background.

Figure 1b shows examples of visual motion stimuli (flow fields). The flow field stimulus was described in detail in Pitzalis et al. (2010). Briefly, participants were instructed to maintain central fixation while presented with eight 16-s blocks of coherently moving dot fields (dilations, contractions, spirals and rotations) interleaved with eight 16-s blocks of randomly moving dot fields. A new field of dots was generated every 500 ms (dot size 0.4 × 0.4 deg^2^). The pattern motion was chosen randomly for that 500-ms period from a continuum ranging from dilation to outward spiral, to rotation, to inward spiral, to contraction. The center of the movement was jittered from flow to flow, and the speed varied within a small range. During the random OFF period, dots and their movement vectors were generated as during the coherent ON periods except that each dot trajectory was rotated by a random angle around the pattern center before execution. This scrambled the coherency of movement (at a given point, dots moved in different directions) but preserved the speed gradient (central dots still moved slower than peripheral dots). The average luminance of the stimulus was 31 cd/m.

A subset of participants also completed an additional low-level visual motion stimulus (radial rings; Fig. 1c) originally used by Tootell et al. (1995) to functionally define the motion area MT. Here, we used it to further explore the motion sensitivity of all tested regions. Participants were instructed to maintain central fixation while presented with eight 16-s blocks of expanding and contracting rings (7 deg/s) on a slightly darker-gray background interleaved with eight 16-s blocks of stationary rings. During motion blocks, the concentric rings periodically contracted and expanded (1 s, 1 s) to avoid generating motion aftereffects during the static blocks (further details in Pitzalis et al. 2010).

We have also performed a psychophysical validation to verify and quantify the motion sensation evoked by the two visual motion stimuli (flow fields and radial rings; see Supplementary Materials and Supplementary Figure 3).

### Experimental setup

For the scene/place experiment, we used a standard setup where the projection screen was attached to the back of the MR bore, and the average viewing distance was 66.5 cm, subtending 19 by 14 deg of visual angle.

For both flow field and radial ring experiments, we used a wide field setup similar to that originally described by our group (Pitzalis et al. 2006, 2010, 2013a, b; Strappini et al. 2015, 2017). Shortly, stimuli were projected onto a back-projection screen attached to the back of the head coil, at a distance of about 21 cm from the subjects’ eyes and seen in binocular view via an enlarged mirror. Subjects’ head was lowered of about 4 cm from isocenter so that even the bottom portion of the screen could be seen. In such conditions, visual stimuli subtended up to 70 by 55 deg of visual angle. Nevertheless, subjects could comfortably fixate a central point on the screen without blurring.

Head movements were minimized with mild restraint and cushioning. Stimuli were generated by a control computer located outside the MR room, and presented using an in-house software based on MATLAB. An LCD video projector with a customized lens projected the visual stimuli to a back-projection screen mounted inside the MR tube and visible through a mirror mounted inside the head coil. Presentation timing was controlled and triggered by the acquisition of fMRI images.

### Image acquisition and processing

MRI images were acquired on a 3T Siemens Allegra MR scanner and on a Philips Achieva 3T at the Santa Lucia Foundation (Rome, Italy) for 102 of the 134 participants who were involved in the study, and on a Philips Achieva 3T scanner at the Institute for Advanced Biomedical Technologies (ITAB) of the University G. D’Annunzio Foundation in Chieti (Italy) for the remaining 32 participants.

Scanners were equipped for echo-planar imaging and a standard head coil was used. Functional T2*-weighted images were collected for the whole cerebral cortex (excluding only the ventral portion of the cerebellum) using a gradient echo EPI sequence using blood-oxygenation level-dependent imaging [Siemens parameters: 30 slices, interleaved excitation order (0 mm gap), in-plane resolution = 3 × 3 mm, slice thickness = 4 mm, repetition time (TR) = 2000 ms, echo time (TE) = 30 ms, image matrix = 64 × 64, flip angle = 70 deg; Philips (Rome) parameters: 38 slices, ascending excitation order (1 mm gap), in-plane resolution = 2.5 × 2.5 mm, slice thickness = 4 mm, TR = 2000 ms, TE = 30 ms, image matrix = 64 × 64, flip angle = 77 deg]. Philips (Chieti) parameters: 39 slices, interleaved excitation order (0 mm gap), in-plane resolution = 3.59 × 3.59 mm, slice thickness = 3.59 mm, TR = 1.869, TE = 25 ms, image matrix = 64 × 64, flip angle = 80 deg].

We also collected a T1-weighted sequence for each participant [Siemens sagittal magnetization-prepared rapid acquisition gradient echo (MPRAGE) sequence: 176 slices, in-plane resolution 0.5 × 0.5 mm, slice thickness = 1 mm, TR = 2 s, TE = 4.38 ms, image matrix = 512 × 512, flip angle = 8 deg; Philips (Rome) turbo field echo (TFE) sequence: 342 slices, in-plane resolution = 0.5 × 0.5 mm, slice thickness = 1 mm, TR = 0.04 s, TE = 5.84 ms, image matrix = 512 × 512, flip angle = 8 deg; Philips (Chieti) MPRAGE sequence: 160 slices, in-plane resolution = 0.5 × 0.5 mm, slice thickness = 1 mm, TR = 2 s, TE = 4.4 ms, image matrix = 512 × 512, flip angle = 8 deg].

Overall, each subject underwent one or two acquisition sessions and completed two 468-s-long scene/place scans (*N* = 61) or two 256-s-long flow field scans (*N* = 96) or both (*N* = 23). Some participants (*N* = 35) completed also two additional 256-s-long radial rings scans.

Structural images were analyzed using FreeSurfer 5.1 (http://surfer.nmr.mgh.harvard.edu/) to obtain a surface representation of each individual cortical hemisphere in a standard space. We used the “recon-all” fully automated processing pipeline, which, among other steps, performs intensity correction, transformation to Talairach space, normalization, skull-stripping, subcortical and white-matter segmentation, surface tessellation, surface refinement, surface inflation, sulcus-based nonlinear morphing to a cross-subject spherical coordinate system, and cortical parcellation (Dale et al. 1999; Desikan et al. 2006). The resulting surface reconstructions were transformed to the symmetrical FS-LR space (Van Essen et al. 2012) using tools in the Connectome Workbench software (https://www.humanconnectome.org/software/get-connectome-workbench), resulting in surface meshes with approximately 74 K nodes per hemisphere.

Functional images were realigned within and across scans to correct for head movement and coregistered with structural images scans using SPM12 (Wellcome Department of Cognitive Neurology, London, UK). Functional data were then resampled to the individual cortical surface using ribbon-constrained resampling as implemented in Connectome Workbench (Glasser et al. 2013) and finally smoothed along the surface with an iterative procedure emulating a Gaussian kernel with a 6-mm full width at half maximum (FWHM).

### Statistical analyses of task-evoked fMRI activity

Hemodynamic responses associated with experimental blocks were estimated according to the general linear model (GLM), as implemented in SPM12. Neural responses during “active” blocks were modeled as box-car functions, convolved with a canonical hemodynamic response function and used as predictors in the GLM, with separate predictors for each condition. In the scene/place stimulus, active blocks included blocks of scenes/places and faces while passive fixation blocks were used as baseline as thus not explicitly modelled as GLM regressors. In the flow field stimulus, active blocks included blocks of coherent moving dots, while blocks of random motion were not explicitly modeled as GLM regressors and, thus, treated as part of residual variance.

We analyzed these tasks on a vertex-by-vertex basis, applying the GLM to the surface-transformed and smoothed images and at regional level, by applying the GLM to regional time courses obtained through averaging the surface-transformed but unsmoothed BOLD time series across nodes in specific regions of interest (ROIs), as detailed below. For the vertexwise analysis, we obtained for each individual hemisphere a parametric map of the *t* statistics, representing the activation during active blocks relative to baseline. Group-level statistical parametric maps were formed through one-sample *t* tests, comparing signal in each condition to the baseline, except for the scene/place stimulus in which a paired *t* test was used to assess the preference for the scene/place condition over the face condition. A conjunction null analysis (Nichols et al. 2005) across the whole brain between the scene/place and flow field stimuli was conducted to find brain regions activated by both scene- and egomotion-related stimuli. To obtain this, parameter estimated images from each participant and stimulus entered a group analysis where subjects were treated as a random effect. The statistical parametric map resulting for this analysis was thresholded at *p* < 0.05 FDR corrected at the cluster level, after applying a cluster-forming threshold of *p* < 0.001 uncorrected at the voxel level.

### Regions of interest (ROIs) definition

We created three set of regions of interest (ROIs) probabilistically defined by averaging individual ROIs from independent samples of participants. We followed standard procedures described in detail below.

1. Egomotion-selective regions. These ROIs were defined by averaging individual ROIs from 18 participants who underwent the flow field localizer in a previous fMRI experiment (see Serra et al. 2019 for further details). As recently reported (Sereno et al. 2001; Pitzalis et al. 2010; Serra et al. 2019), egomotion-selective regions were the regions responding stronger to coherent than to random motion. Six distinct cortical regions are strongly and bilaterally activated by the flow field stimulus: (1) the V6 complex (or V6+) in the dorsal part of the parieto-occipital sulcus (POS), which includes areas V6 and, anteriorly, V6Av (Pitzalis et al. 2013b; Tosoni et al. 2015); (2) the ventral portion of the posterior intraparietal sulcus (pIPS), mainly including the dorsal portion of the retinotopically defined V3A (Pitzalis et al. 2010; Sereno et al. 2001), that we call pIPS/V3A; (3) the intraparietal motion area, known as IPSmot (Pitzalis et al. 2013c), in the horizontal segment of the IPS, likely corresponding to the human VIP (see Huang and Sereno 2018 for a recent review); (4) the cingulate sulcus visual area (CSv), in the depth of the posterior part of the cingulate sulcus, anterior to the posterior ascending portion of the cingulate sulcus, corresponding to the original motion area described by Wall and Smith (2008); (5) the posterior cingulate sulcus area (pCi), within the posterior dorsal tip of the cingulate sulcus (Serra et al. 2019), corresponding to the precuneus motion area (Pc) originally described by Cardin and Smith (2010); (6) the posterior insular cortex (PIC), at the junction between the posterior insula and the posterior parietal cortex (see Greenlee et al. 2016 for a review).

2. Scene-selective regions. These ROIs were defined by averaging individual ROIs from 44 participants who underwent the scene/place localizer in previous fMRI experiments (Sulpizio et al. 2013, 2014, 2016a, 2018 for further details). As previously reported (Sulpizio et al. 2013, 2018), scene-selective regions were the cortical areas responding stronger to pictures of scenes/places than to pictures of faces. Four different cortical regions are strongly and bilaterally activated by the scene/place stimulus: (1–2) the parahippocampal place area (PPA) in the posterior parahippocampal cortex, which include two distinct foci of activation along the posterior–anterior axis (pPPA and aPPA), consistently with previous reports (Baldassano et al. 2013, 2016); (3) the retrosplenial complex (RSC), in the retrosplenial/parieto-occipital sulcus, at the junction with the anterior calcarine sulcus, and (4) the occipital place area (OPA) near the transverse occipital sulcus.

3. Motion-selective area MT+. This ROI was defined by averaging individual ROIs from 19 participants who underwent the radial ring stimulus in previous fMRI experiments (Pitzalis et al. 2012, 2013d). According to a previous study (Tootell et al. 1995) and also as routinely done in our laboratory (e.g., Pitzalis et al. 2010, 2012, 2013a, b, c), MT+ was functionally defined on the cortical surface of each participant as the set of all contiguous cortical nodes within the cortical portion between the Inferior Temporal sulcus (ITs) and the Middle temporal Sulcus (MTs).

All these ROIs were created on each individual hemisphere of the ROI-defining samples, applying the GLM to the surface-transformed smoothed fMRI images. The contrast maps (coherent > random for egomotion-selective regions, scenes/places > faces for scene-selective regions and motion > static for motion-selective area MT +) were corrected for multiple comparisons at the cluster level (*p* < 0.05) through a topological false discovery rate procedure based on random field theory (Chumbley et al. 2010), after defining clusters of adjacent vertices surviving at least an uncorrected voxel-level threshold of *p* < 0.001. Each individual ROI was then selected by isolating single activation peaks and their neighborhood through a watershed segmentation algorithm as applied to surface meshes (Mangan and Whitaker 1999).

**Fig. 2:**
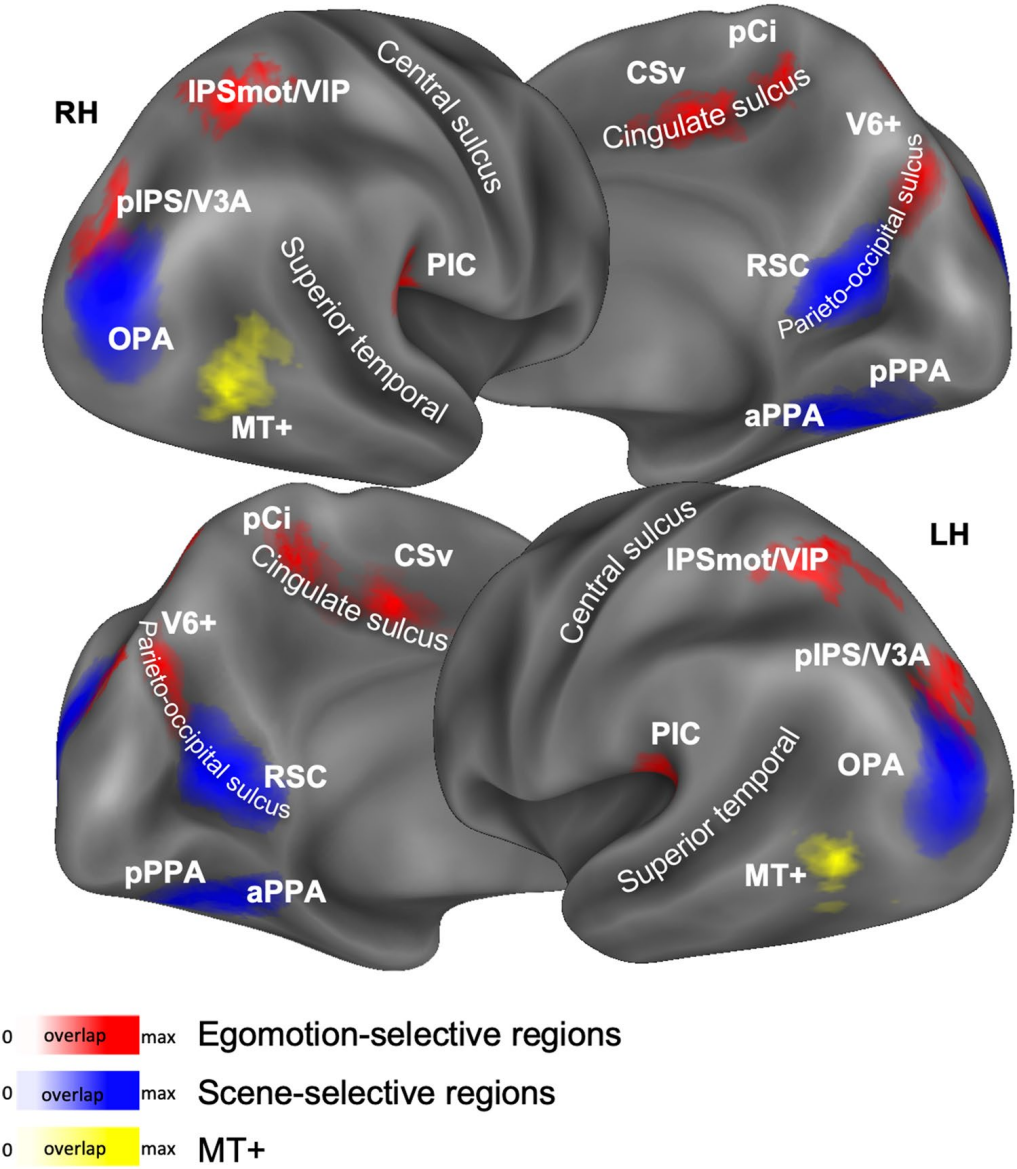
Brain location of the probabilistically defined regions. Egomotion-selective V6+, pIPS/V3A, IPSmot/VIP, CSv, pCi, PIC (in red), scene-selective aPPA, pPPA, RSC, OPA (in blue) and MT+ region (in yellow) are overlapped onto Conte69 brain atlas in different views (lateral and medial) of both left (LH) and right (RH) hemispheres. The color saturation represents the proportion of participants whose region included that node: the higher the color saturation, the higher the probability that the node belongs to the corresponding region. Regions are labeled as followed: *V6+* V6 complex, *IPSmot/VIP* intraparietal motion area/ventral intraparietal, *pIPS/V3A* posterior intraparietal sulcus/V3A, *CSv* cingulate visual area, *pCi* posterior cingulate sulcus area, *PIC* posterior insular cortex, *aPPA* anterior parahippocampal place area, *pPPA* posterior parahippocampal place area, *RSC* retrosplenial complex, *OPA* occipital place area, *MT+* MT complex

Figure 2 shows the anatomical location of the probabilistic egomotion-selective (red), scene-selective (blue) regions and MT+ region (yellow) overlaid onto the inflated Conte69 atlas surface (Van Essen et al. 2012).

### Regional analysis

The main corpus of the analysis was conducted on two set of ROIs, i.e., egomotion- and scene-selective regions.

To test the presence of scene-related responses in egomotion-selective areas (V6+, pIPS/V3A, IPSmot/VIP, CSv, pCi, PIC) and the presence of egomotion-related responses in scene-selective areas (aPPA, pPPA, RSC, OPA), we interrogated the probabilistically defined egomotion-selective regions with respect to the scene/place stimulus and the probabilistically defined scene-selective regions with respect to the flow field stimulus. For each ROI, each node was assigned a weight equal to the proportion of subjects in the ROI-defining sample who showed activation in that node: these values were used to weight the contribution of each node during the extraction of regional signals. For each participant and region, we computed a regional estimate of the amplitude of the hemodynamic response, obtained by entering the weighted spatial average of the pre-processed time series into the individual GLMs. We first computed the percent signal change for coherent > random motion in the flow field stimulus and for scenes > faces in the scene/place stimulus. Regional hemodynamic responses were thus analyzed through a series of one sample *t*-tests, assessing the presence of scene-related response in egomotion-selective regions, and the presence of egomotion-related response in the scene-selective regions.

Additionally, we tested the responsiveness of all these regions to a low-level visual motion stimulus (radial rings), the stimulus originally used to identify the human motion middle temporal area (MT complex or MT+; Tootell et al. 1995), but also able to activate the posterior portion of the retinotopic V3A, as observed by Sereno et al. (2001) and Pitzalis et al. (2010). We explored the response of both the egomotion- and scene-selective regions to this radial motion stimulus to verify a possible different function profile of these regions in terms of basic motion sensitivity. Since this stimulus does not induce a self-motion sensation (see Supplementary Materials and Supplementary Figure 3) and, therefore, does not represent a navigationally relevant motion cue, we expected to find a response only in regions involved in the low-level processing of visual motion. Similar to the main tasks, radial rings blocks were modeled as box-car functions, convolved with a canonical hemodynamic response function. Active blocks included blocks of moving rings while passive blocks included blocks of stationary rings, and thus treated as part of residual variance. We, thus, subjected individual regional parameter estimates representing signal changes in the motion condition to one-sample *t* tests versus zero to reveal regions showing a low-level radial motion sensitivity.

Further analyses were conducted to establish the role of MT+ during both motion processing and scene/place perception. Following the above-described procedure, regional hemodynamic responses were thus analyzed through a series of one sample *t*-tests, assessing the presence of egomotion- and scene-related response in area MT+.

Finally, since we observed some degree of overlap between the probabilistically defined scene-selective OPA and the egomotion-selective pIPS/V3A (Fig. 2), we performed further regional analyses to better define the relative location of these two regions at an individual level. We, thus, defined a new set of individual ROIs by isolating scene-selective OPA and motion-selective pIPS/V3A on the left and right hemispheres of each single subject of the present study. First, we identified these two regions applying the GLM to the surface-transformed smoothed fMRI images and using the same contrasts (OPA: scenes > faces; pIPS/V3A: coherent > random) previously used for the probabilistically defined ROIs. Then, individual ROIs were selected from the resulting statistical maps, using a threshold-free mapping, by selecting single activation peaks and their neighbourhood (for a maximum of 400 cortical nodes) through a watershed segmentation algorithm as applied to surface meshes (Mangan and Whitaker 1999). We, thus, inspected the relative positions of OPA and pIPS/V3A in each individual hemisphere and checked the degree of overlap between these regions, by quantifying the number of common nodes between them. We then isolated the cortical nodes corresponding to this common area and subtracted it from OPA and pIPS/V3A. Following this procedure, we defined in each individual hemisphere three distinct regions classified as: (1) OPA-only, in which any cortical node shared with pIPS/V3A was removed, (2) common, including only cortical nodes belonging to both OPA and pIPS/V3A and (3) pIPS/V3A-only, in which any cortical node shared with OPA was removed.

Next, for each subject and region, we computed Pearson correlations across nodes between the percent signal changes evoked by our stimuli, to test for the interdependence between egomotion- and scene-related activity, and between these activations and those elicited by the low-level visual motion stimulus (radial rings). After transforming correlations coefficients to z-values using the Fisher transform, we performed a series of one-sample *t*-tests against zero to reveal the existence of significant positive correlations, indicating a relationship between the task-related activities. Finally, following the same procedure previously described for the probabilistically defined regions (see above), we tested the motion selectivity of OPA-only, common and pIPS/V3A-only and their selectivity to the low-level visual motion stimulus (radial rings), through a series of one sample *t*-tests.

For all the above-mentioned analyses, a Bonferroni correction was applied to account for multiple comparisons (*p* = 0.05/*N* = number of regions).

## Results

### Scene-related responses in egomotion-selective regions, and egomotion-related responses in scene-selective regions

To test whether egomotion-selective regions are sensitive to navigationally relevant information in the absence of visual motion, and whether scene-selective regions are sensitive to coherent visual motion in the absence of navigational scene, we analyzed the response profile of V6+, pIPS/V3A, IPSmot/VIP, CSv, pCi, PIC during the scene/place stimulus and the response profile of aPPA, pPPA, RSC, OPA during the flow field stimulus, respectively.

**Fig. 3:**
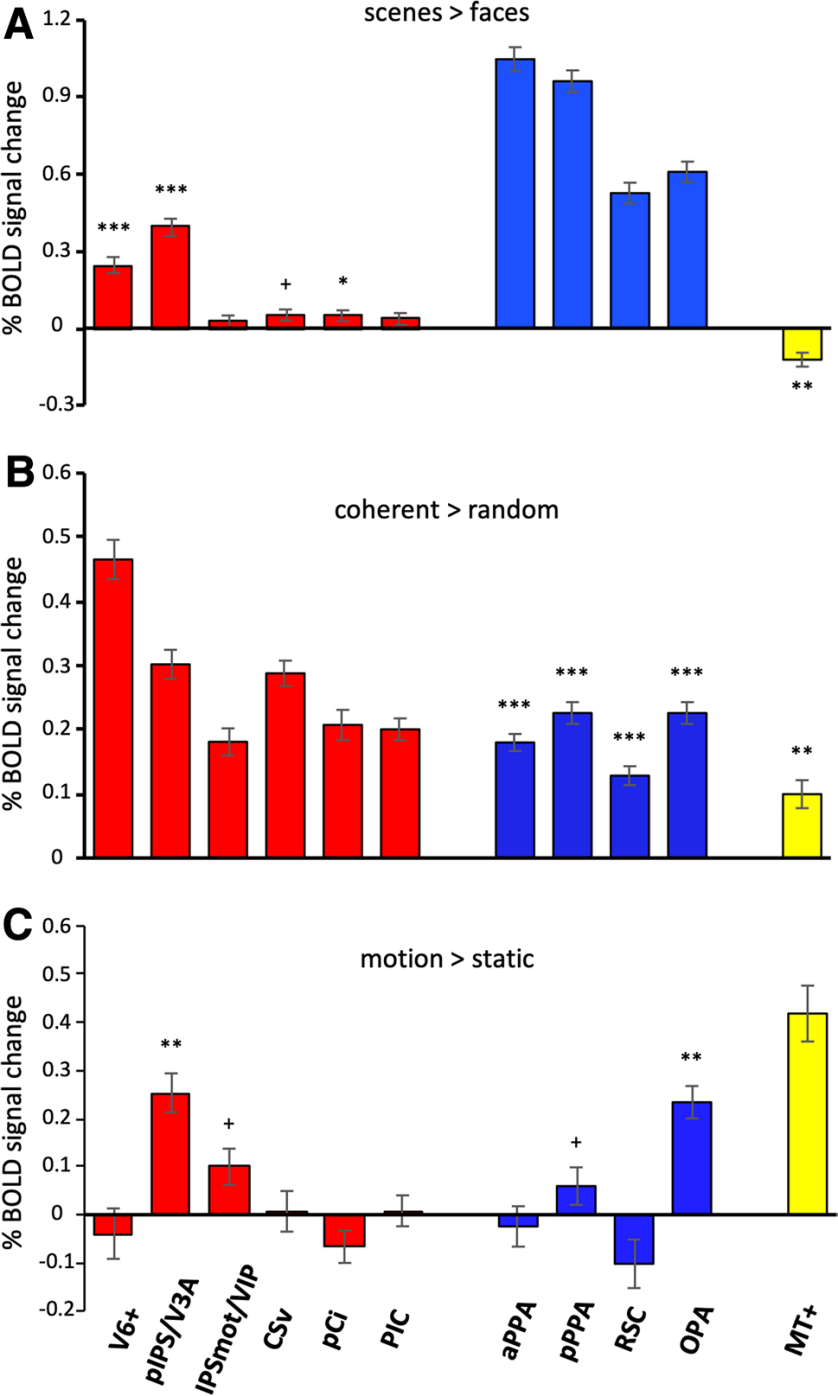
Regional analyses. Plots show the mean percentage of BOLD signal change (± standard error) of the egomotion-selective regions (V6+, pIPS/V3A, IPSmot/VIP, CSv, pCi, PIC), the scene-selective regions (aPPA, pPPA, RSC, OPA) and MT+ during the scenes > faces contrast of the scene/place stimulus (**a**), the coherent > random contrast of the flow field stimulus (**b**) and the motion > static contrast of the radial ring stimulus (**c**). ***p < 10^−8^ (Bonferroni corrected); **p < 10^−5^ (Bonferroni corrected); *p < 0.005 (Bonferroni corrected); ^+^*p* < 0.05 (Bonferroni uncorrected). Regions labels as in Fig. 2

**Fig. 4:**
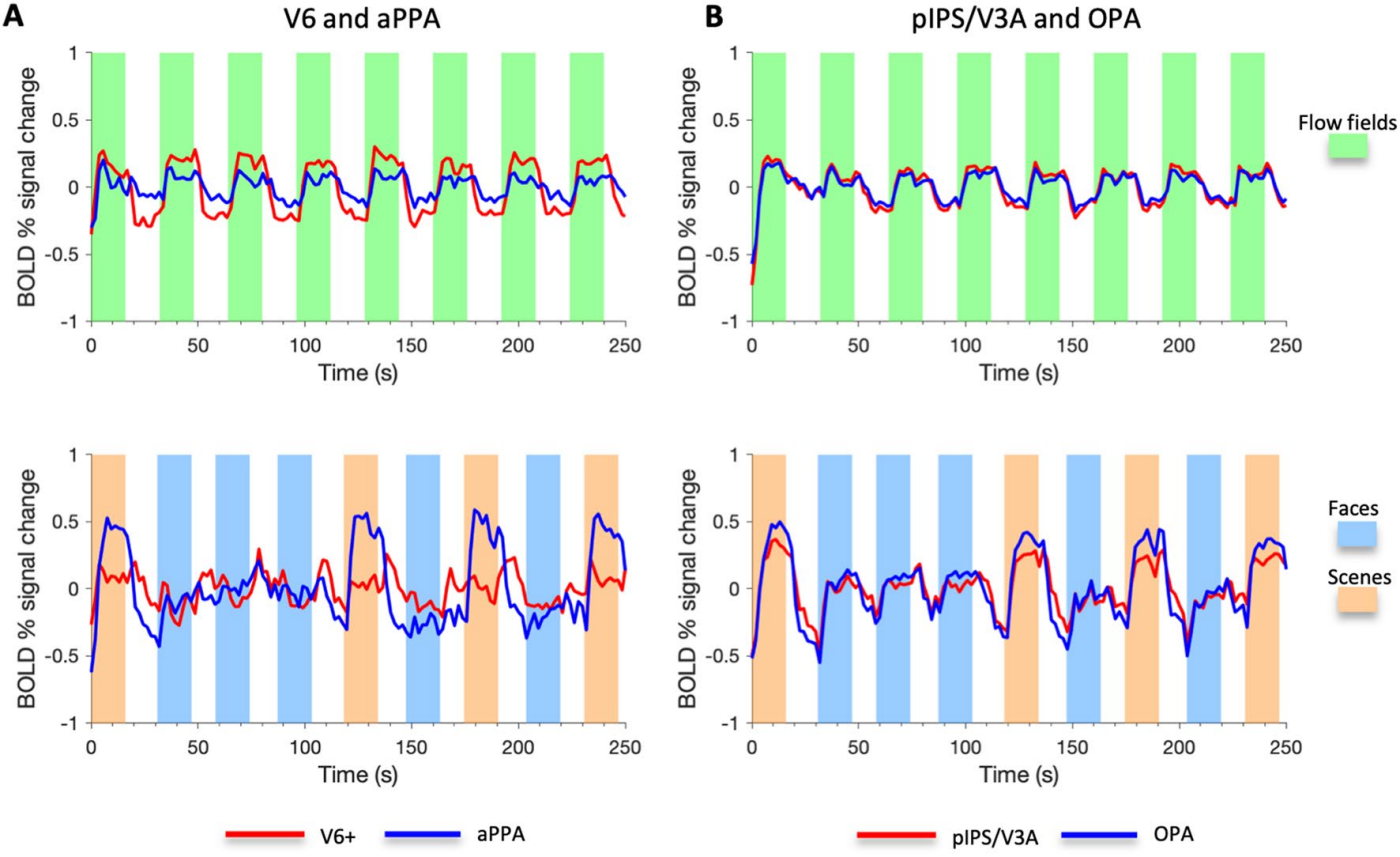
Time courses of representative egomotion- and scene-selective ROIs. **a** Across-scans and across-subjects average of activity of V6+ (red line) and aPPA (blue line), i.e., the two most responsive regions to egomotion- and scene-related information, respectively, is shown as a function of time (first 250 s) for both flow field (upper panel) and scene/place scans (lower panel). **b** The same for pIPS/V3A (red line) and OPA (blue line), i.e., the two regions showing functional and anatomical similarities (see also Fig. 7)

Plots in Fig. 3a, b show the percent signal change (across the two hemispheres) for scenes > faces and for coherent > random in egomotion- (red bars) and scene-selective (blue bars) regions, respectively. For the scene/place stimulus (Fig. 3a), V6+ and pIPS/V3A strongly preferred scenes to faces (left hemisphere: V6+: *t*_60_ = 7.56, *p* = 2.75 × 10^−10^; pIPS/V3A: *t*_60_ = 10.25, *p* = 8.25 × 10^−15^; right hemisphere: V6+: *t*_60_ = 7.76, *p* = 1.27 × 10^−10^; pIPS/V3A: *t*_60_ = 11.97, *p* = 1.52 × 10^−17^). To further characterize the functional profile of these regions we also showed their time courses during both flow field and scene/face stimuli. Figure 4a shows the V6+ time course during both flow field and scene/face stimuli in comparison with that of aPPA, which is the most activated region by the scenes > faces contrast. Although the time courses of these areas were pretty similar during the flow field stimulus, a qualitative comparison of the V6+ and aPPA time courses during the scene/place localizer revealed several differences. In particular, V6+ seemed to have a general deactivation during blocks of faces and a feeble/null response during blocks of scenes/places, while aPPA showed the expected peak of activation, followed by a sustained activity, only during blocks of scenes. In other words, although V6+ exhibited a preference for scenes as compared to faces, this result cannot be taken as a strong evidence of scene-related response. Figure 4b also shows the time course of pIPS/V3A in comparison with that of OPA. In this case, the pIPS/V3A preference for scene blocks reflected a genuine sensitivity to scene-related information, being its time course during scene/face blocks very similar to that observed in the scene-selective OPA.

A significant response during the scene/place stimulus was also observed in the right pCi (*t*_60_ = 3.04, *p* = 0.005), and a significant, but Bonferroni-uncorrected, response was found in the bilateral CSv (left: *t*_60_ = 2.60, *p* = 0.012; right: *t*_60_ = 2.58, *p* = 0.012). However, the inspection of regional time course revealed the absence of a genuine scene-related response in these regions, being their preference for the scenes > faces contrast mainly explained by a stronger deactivation during the face blocks (Supplementary Figure 2). For the flow field stimulus (Fig. 3b), all scene-selective regions showed a significant strong positive response (left hemisphere: aPPA: *t*_95_ = 11.76, *p* = 3.06 × 10^−20^; pPPA: *t*_95_ = 11.71, *p* = 4.04 × 10^−20^; RSC: *t*_95_ = 7.87, *p* = 5.22 × 10^−12^; OPA: *t*_95_ = 12.14, *p* = 4.95 × 10^−21^; right hemisphere: aPPA: *t*_95_ = 13.99, *p* = 8.11 × 10^−25^; pPPA: *t*_95_ = 13.94, *p* = 1.07 × 10^−24^; RSC: *t*_95_ = 9.05, *p* = 1.80 × 10^−14^; OPA: *t*_95_ = 13.14, *p* = 4.35 × 10^−23^). Time courses of all regions showing significant effects are provided in the Supplementary Figure 2.

Although we formally tested the sensitivity of the two groups of regions only with respect to the non-preferred stimulus category, we also showed the percentage of signal change of each region during both flow field and the scene/place stimuli (see Fig. 3a, b). This allowed us to highlight the relative strength of activations between egomotion- and scene-selective regions during both preferred and non-preferred stimuli. Note that any comparison between the two set of ROIs is merely illustrative, due to the lack of independency between the data and the selection criteria. Figure 3a showed the response to the scene/place stimulus in both scene- and egomotion-related regions. As expected, we observed a stronger activation in all the scene-selective regions, and in PPA in particular, as compared to all egomotion-selective regions. Figure 3b showed the response to the flow field stimulus in both scene- and egomotion-related regions. As expected, the response to the flow field stimulus was particularly strong in V6+. With respect to the scene-selective regions, we observed higher activation in CSv and pIPS/V3A, but a comparable response in IPSmot/VIP, pCi and PIC. This can be explained by considering that the flow field is a powerful stimulus able to activate different brain regions, although at different extent. As previously showed in Pitzalis et al. (2019), the flow field stimulus activates a wide network of areas which includes not only the above-described high-level egomotion regions, but also the mesial, anterior part of the occipital lobe, such as the boundary between the posterior parahippocampal cortex and the anterior lingual gyrus and the conjunction between the calcarine sulcus and the parietal-occipital sulcus, where PPA and RSC are, respectively, located. However, the individual inspection of hemisphere-specific activation maps did not reveal consistent foci of activation in the cortical territory of PPA and RSC (Serra et al. 2019). Additionally, the spots of activation found in the posterior segment of the IPS and labelled as pIPS/V3A in the ROI-defining sample likely cover the OPA territory (see Serra et al. 2019 for inspection of some individual maps). This might explain why the activation found here in OPA is comparable to that observed in some egomotion-selective regions and support the possibility that pIPS/V3A and OPA are part of a unique functional complex (see below).

To further explore the motion selectivity in the two classes of regions, we analyzed their response profile during the low-level visual motion stimulus (radial rings), comparing moving with stationary stimuli. Figure 3c shows the percent signal change (across the two hemispheres) for the motion > static comparison. We found that, among all the scene- and egomotion-selective regions, only OPA (left: *t*_34_ = 5.90, *p* = 1.16 x 10^−6^; right: *t*_34_ = 7.34, *p* = 1.65 × 10^−8^) and pIPS/V3A (left: *t*_34_ = 6.41, *p* = 2.52 × 10^−7^; right: *t*_34_ = 6.03, *p* = 7.97 × 10^−7^) were significantly activated by the motion rings. Significant, but Bonferroni-uncorrected, response was observed in the bilateral IPSmot/VIP (left: *t*_34_ = 2.84, *p* = 0.007; right: *t*_34_ = 2.61, *p* = 0.013) and in the right pPPA (*t*_34_ = 2.08, *p* = 0.045).

### Scene- and egomotion-related responses in MT+

We also characterized the role of area MT+, classically considered the key motion region of the dorsal visual stream (Tootell et al. 1995; Morrone et al. 2000; Smith et al. 2006; Cardin and Smith 2010; see also Pitzalis et al. 2013a for a review) with respect to both scene/place and flow field stimuli. Plots in Fig. 3a, b also show the percent signal change (across the two hemispheres) for scenes > faces and for coherent > random in area MT+ (yellow bars). For the scene/place stimulus (Fig. 3a), this motion area showed a significant negative response (left hemisphere: *t*_60_ = − 3.58; *p* = 6.89 × 10^−4^; right hemisphere; *t*_60_ = − 5.28; *p* = 1.91 × 10^−6^), indicating a stronger response to faces as compared to scenes/places. For the flow field stimulus (Fig. 3b), MT+ showed a significant positive response (left hemisphere: *t*_95_ = 2.96; *p* = 3.94 × 10^−3^; right hemisphere; *t*_95_ = 6.26; *p* = 1.11 × 10^−8^) indicating a preference for the coherent as compared to the random motion. For comparison, we also showed the response of MT+ to the low-level visual motion stimulus (radial rings; Fig. 3c), although a quantitative comparison between this area and both egomotion- and scene-selective regions is not strictly appropriate.

These results confirm the primary role of MT+ in the analysis of motion signals (see Galletti and Fattori 2003 for a review). In a first paper, we showed that the flow field stimulus does not elicit statistically significant responses in MT+ (see, e.g., Pitzalis et al. 2010). In a more recent paper, we showed that MT+ shows some degree of preference for egomotion-compatible optic flow, although with a weak consistency across subjects (lower than 70%; Serra et al. 2019). Cardin and Smith (2010) found significant responses to egomotion-compatible optic flow in MT+, although selectivity in this area was not as pronounced as in VIP and CSv (Wall and Smith 2008). Here, we confirmed our previous observations of a moderate MT+ preference for the coherent flow field stimulus. In addition, unlike V6+, MT+ showed a preference for faces compared to scenes/places (Fig. 3a). These results go along the same direction of our previous studies showing different functional responses in V6+ and MT+ (Pitzalis et al. 2010, 2013b, 2020).

### Conjunction analysis between scene/place and flow field stimuli

**Fig. 5:**
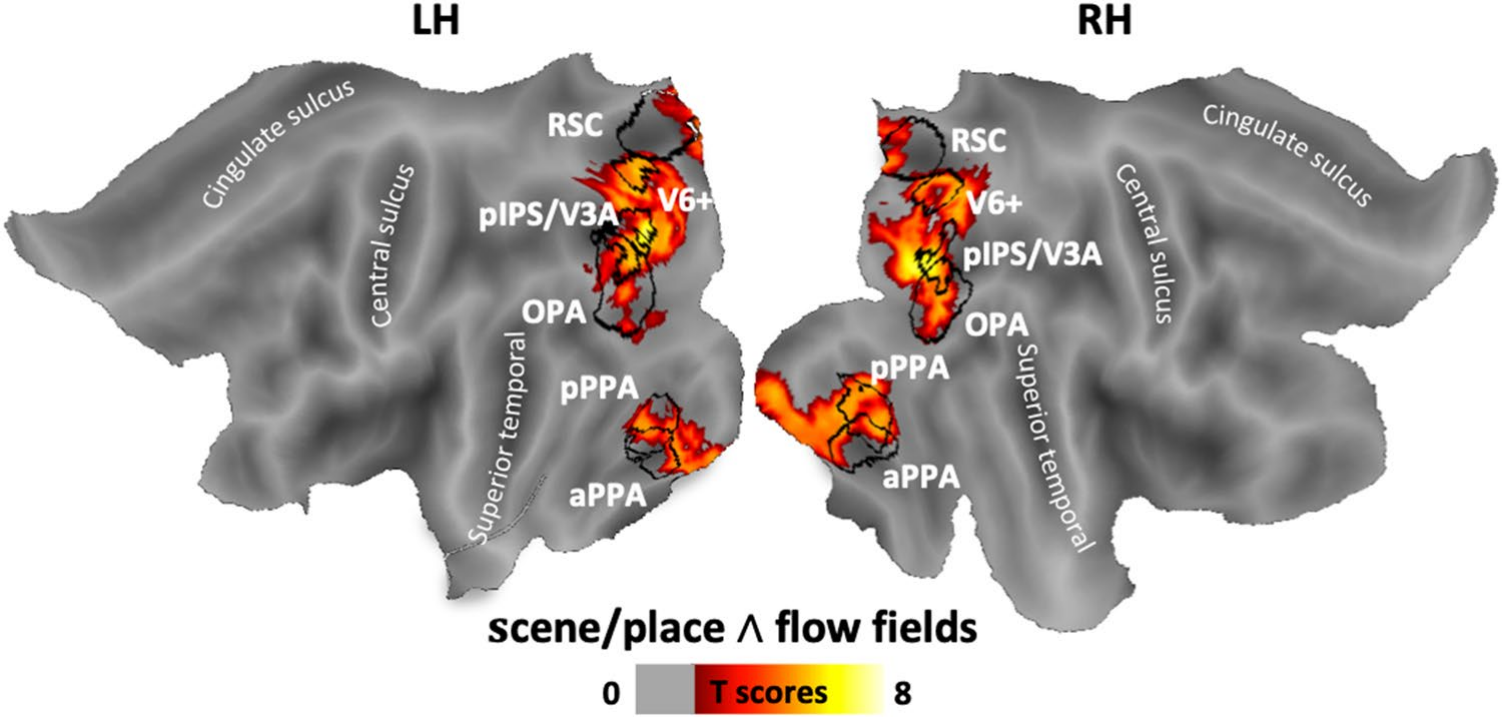
Conjunction analysis. Group-based activation as resulting from the conjunction analysis between scene/place and flow field stimuli superimposed over the flat representation of Conte69 atlas (Van Essen et al. 2012). The borders of the regions of interest touched by the activation (aPPA, pPPA, RSC, OPA, V6+, pIPS/V3A) are marked in black

**Fig. 6:**
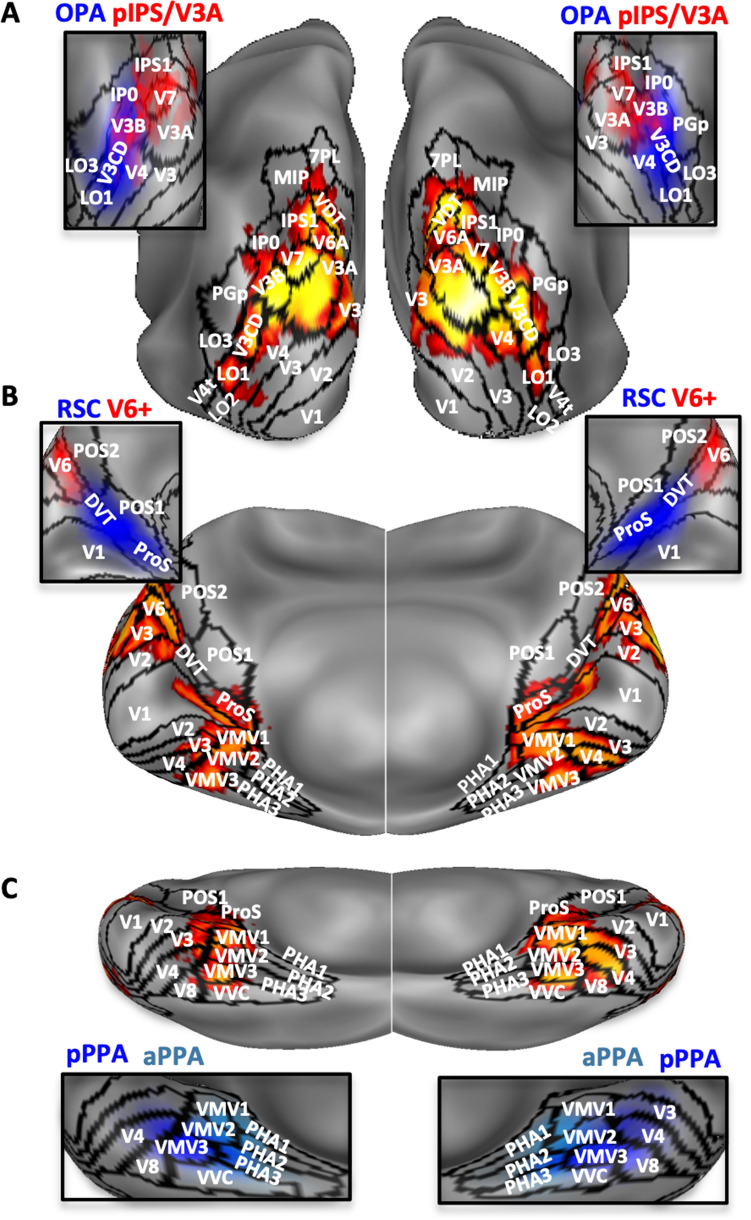
Relationship between the conjunction map and previously defined retinotopic and functional areas. **a** Activation map superimposed over the inflated representation is shown in the posterior view but also in medial (**b**), and inferior (**c**) views. The borders of 31/180 areas coming from a recent multimodal parcellation of the human cortex (Glasser et al. 2016) are indicated using solid black lines. Close-up views show the spatial relationship between the multimodal parcellation and the above-mentioned regions of interest, i.e., OPA and pIPS/V3A (**a**), V6+ and RSC (**b**), aPPA and pPPA (**c**). Regions labels as in Fig. 2. Color bar as in Fig. 4

Figure 5 shows the results of a conjunction null analysis showing the cortical regions activated by both scenes/places and flow fields on a flattened cortical surface reconstruction of the left and right hemisphere of a standard brain. Notice that the activated region overlaps completely with V6+, pIPS/V3A, pPPA, and OPA (black outlines), and only partially with aPPA and RSC. Figure 6 shows the relationship between the conjunction map and the multimodal parcellation of specific retinotopically organized cortical areas from the Human Connectome Project (Glasser et al. 2016). In the close-up (black boxes) of Fig. 6a–c, we also compared the anatomical location of probabilistically defined egomotion- and scene-selective regions with the above-mentioned parcellation.

This analysis revealed common activation for the two stimuli in a bilateral network of areas within the parieto-occipital and the temporo-occipital cortex. In particular, we found a prominent focus of activation within the dorsolateral parieto-occipital cortex (Fig. 6a), in correspondence of the posterior segment of the intraparietal sulcus (pIPS). This activation extended into multiple atlas-based maps, such as V7, V3A, V3B, V3CD, the dorsal portion of both V3 and V4, and more ventrally so as to include the visual fields maps LO1, LO2 (Larsson and Heeger 2006; Sayres and Grill-Spector 2008). Note that the probabilistic egomotion-selective pIPS/V3A (see red spot in the close-up of Fig. 6a), is not restricted to the atlas-based V3A but includes portions of other atlas-based areas as V3B, V7, and IPS1, thus highlighting a lack of a clear consistency between pIPS/V3A and the atlas, especially in the left hemisphere. However, because this atlas is based on a multimodal map obtained combining cytological architecture, functional specialization, connectivity and topographic organization rather than on strictly retinotopic or functional data, the descriptions of the overlay between our probabilistic regions and areal borders from this parcellation should be considered with caution. Although here we did not use retinotopic mapping to define the region, we refer to V3A based on our previous retinotopic studies showing that the pIPS activation found with the flow field stimulus is mainly coinciding with the dorsal part of the retinotopic area V3A (Sereno et al. 2001; Pitzalis et al. 2010).

Interestingly, common activation also included the OPA (see blue spot in the close-up of Fig. 6a), which in turn overlaps with multiple atlas-based maps, including IP0, V3B, V3CD, V4, and visual fields map LO1. Note that pIPS/V3A and OPA are partially overlapped in correspondence of V7 and V3B (see next paragraph).

As shown in Fig. 6b, the parieto-occipital activation extended medially and dorsally within the dorsal margin of the parieto-occipital sulcus (POS), thus including the atlas-based V6 and V6A (Pitzalis et al. 2010, 2013b), and the neighboring V2 and V3, in correspondence of their peripheral representation of the lower visual field. The parieto-occipital activation extended more ventrally along the POS, up to the junction with the calcarine fissure, so as to include the adjacent periphery of V1 and V2, the adjoint prostriate area (Pros) and two newly defined cortical regions as POS1 and visual transitional area (DVT) (Glasser et al. 2016). A more ventral activation (Fig. 5c) was found in correspondence of the ventromedial visual areas 1–3 (VMV1, VMV2, VMV3; Glasser et al. 2016), previously known as parahippocampal areas PHC-1, PHC-2 (Arcaro et al. 2009; Wang et al. 2015) and the ventral occipital VO-2 (Brewer and Barton 2012), respectively. This activation (more clearly visible in Fig. 6c) also extended posteriorly into the far peripheral representation of V2 and V3, and inferiorly into the adjacent V4 and V8. Note that while the V6+, as defined in our study (see red spot in the close-up of Fig. 6b), strictly corresponds to the atlas-based V6, the RSC (blue spot in the close-up of Fig. 6b) includes portions of other areas such as Pros, DVT and POS1. The aPPA (light blue spot in the close-up of Fig. 6c) is mainly centered on the parahippocampal areas PHA1, PHA2, PHA3 (Glasser et al. 2016) and VMV1, while the pPPA (dark blue spot in the close-up of Fig. 6c) includes portions of VMV2 and VMV3 and the most anterior parts of V3, V4, and V8.

Overall, the conjunction analysis first confirms the results of the regional analysis, indicating that the two most posterior egomotion-selective regions (V6+, pIPS/V3A) and all the scene-selective regions (aPPA, pPPA, RSC and OPA) were engaged during processing of both scene- and egomotion-relevant information (see Fig. 4a for V6+ data interpretation). In addition, the comparison between the conjunction analysis, the multimodal parcellation of cortical areas by Glasser et al. (2016), and the anatomical location of probabilistically defined egomotion- and scene-selective areas mapped in the present study shows that the most important focus of common activation is located in the dorsolateral parieto-occipital cortex, in a cortical territory hosting both OPA and pIPS/V3A.

### Individual inspection of scene-selective OPA and egomotion-selective pIPS/V3A

To better evaluate the degree of overlap between OPA and pIPS/V3A, we defined these two cortical regions on the individual subjects of the current study (*N* = 23) who participated to both flow field and scene/place experiments. In this way, we were able to examine the relative position of OPA and pIPS/V3A at the individual level, i.e., to reliably check the extent of the overlap of the two regions on the cortical surface of the same subject.

We successfully identified OPA and pIPS/V3A in all subjects participating to both experiments (46/46 hemispheres). According to the location of the probabilistic ROI (see Fig. 2), OPA was located at the intersection between the pIPS and TOS and pIPS/V3A was found in the ventral portion of the posterior intraparietal sulcus (pIPS), at the junction with the temporal occipital sulcus (TOS). In line with the position of the probabilistic pIPS/V3A (see Fig. 2), the individual pIPS/V3A extended more dorsally and medially with respect to neighboring OPA. More specifically, with respect to OPA, pIPS/V3A was located medially in 13/46 hemispheres, dorsomedially in 19/46 hemispheres, dorsally in 12/46 hemispheres, and laterally in 1/46 hemispheres. In only 1/46 hemisphere the two regions were almost totally coincident. The two regions shared common nodes in 39/46 hemispheres, with different degrees of overlap varying from a minimum of 5/400 nodes (1%) to a maximum of 175/183 nodes (95%). The overlap was more prominent in the right hemisphere (23/23 participants) and less in the left hemisphere (16/23 participants).

**Fig. 7:**
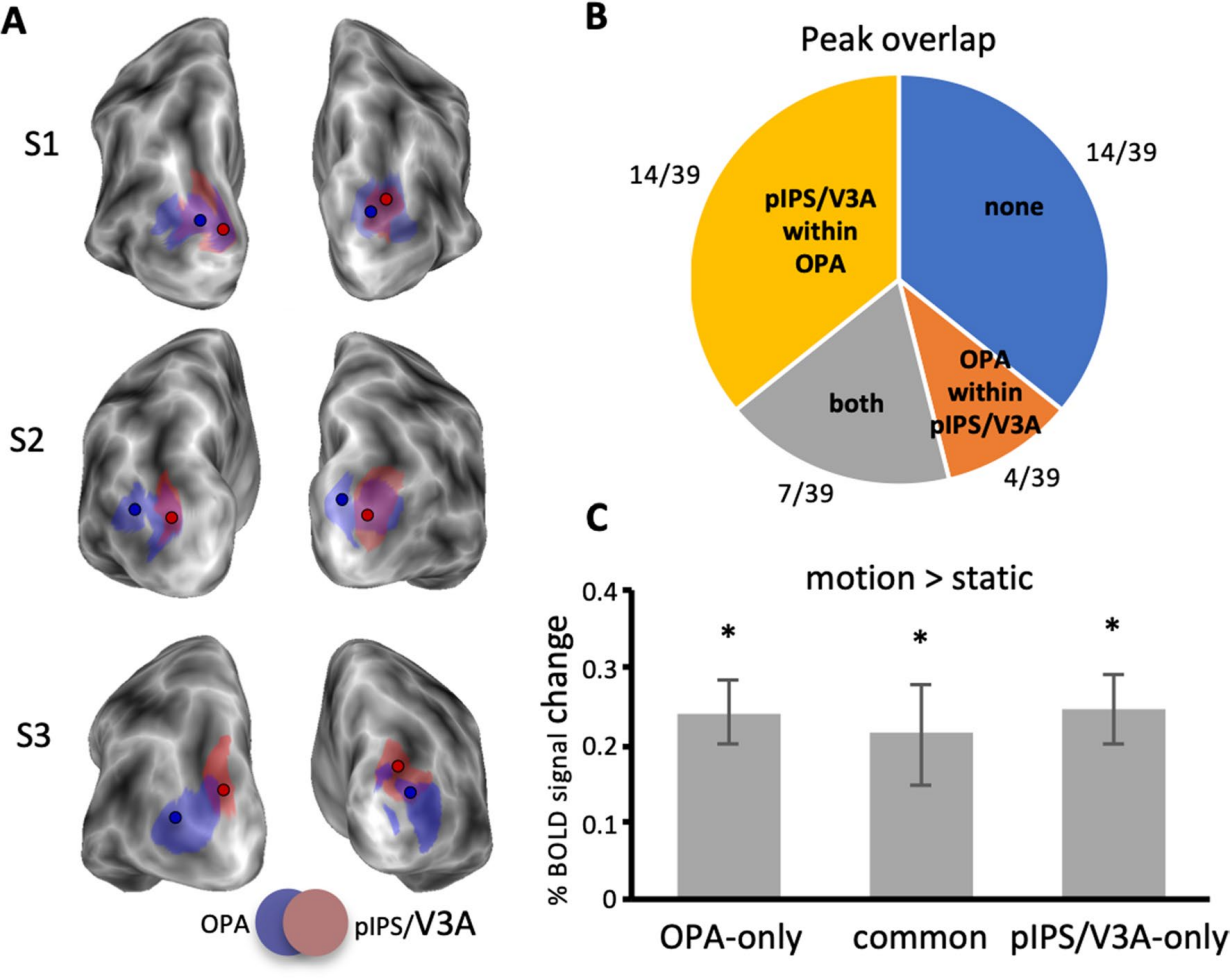
OPA vs. pIPS/V3A relationship. **a** The relative location of bilateral OPA and pIPS/V3A and the overlapping extent are displayed on the cortical surface reconstruction of three representative participants (S1–S2–S3). **b** Group-average frequency of peak overlap between the two regions, collapsed across hemispheres. Four different scenarios are observed: (1) pIPS/V3A peak falling into the OPA boundary, (2) OPA peak falling into the pIPS/V3A boundary, (3) pIPS/V3A peak falling into the OPA boundary and vice versa (both), and (4) no overlap (none). **c** Plot shows the mean percentage of BOLD signal change (± standard error) of the OPA-only, common and pIPS/V3A-only regions as a function of the motion > static contrast of the low-level visual motion (radial rings) stimulus. *p < 0.001 (Bonferroni corrected)

Figure 7a shows the relative location of OPA and pIPS/V3A, and the overlapping extent, in three representative participants. We also computed the frequency with which the peak of pIPS/V3A (or OPA) fell into the boundary of the other region (Fig. 7b). In the 39/46 hemispheres hosting overlapping regions, we observed the presence of peak overlap (i.e., the peak of pIPS/V3A falling into the OPA territory, the peak of OPA falling into the pIPS/V3A territory, or both) in 25 hemispheres (64%), while no peak overlap was observed in the remaining 14 hemispheres (36%). We observed that the peak of pIPS/V3A fell within the OPA boundary in the majority of cases (14/39 hemisphere; an example in S2, both hemispheres, is shown in Fig. 7a), while the peak of OPA fell within the pIPS/V3A territory only in 4/39 hemispheres (an example is shown in Fig. 7a: right hemisphere of S3). Both pIPS/V3A and OPA peaks fell into the cortical territory of the other region in 7/39 hemispheres (see an example in Fig. 7a: both hemispheres of S1). Figure 7a also shows an example of no peak overlap (left hemisphere of S3). Overall, in the majority of hemispheres containing overlapping regions, we observed the presence of at least one regional peak falling into the territory of the other region, with the OPA boundary including the peak of pIPS/V3A most frequently.

To better characterize the response profile of these regions, we isolated distinct areas in each individual hemisphere, corresponding to the region including only OPA surface nodes with no nodes belonging to pIPS/V3A (OPA-only), the region including only pIPS/V3A surface nodes with no nodes belonging to OPA (pIPS/V3A-only) and, when present, the region in between including nodes belonging to both OPA and V3A (common). To test how much the activity of these areas depend on both egomotion- and scene-related processing, Pearson correlation coefficients between the activity evoked by the two stimuli were computed across cortical nodes, separately for each hemisphere, and used as an index of interdependence between egomotion- and scene-related activity. Plots in Supplementary Figure 1A show the node-to-node correlations. We found significant positive correlations in all these areas (OPA-only: *t*_45_ = 4.18; *p* = 1.30 × 10^−4^; common: *t*_38_ = 5.18; *p* = 7.04 × 10^−6^, pIPS/V3A-only: *t*_45_ = 5.06; *p* = 11.11 × 10^−6^), indicating that all of them showed a positive relationship between the activity evoked by the two stimulus categories: cortical nodes exhibiting egomotion-related activity also showed scene-related activity These results provide no evidence for separate cortical regions that are sensitive to either egomotion- or scene-related information, thus suggesting that the same population of nodes may encode both visual information.

We also conducted other node-to-node correlations to compute the index of interdependence between the activity evoked by the two motion stimuli (flow fields and radial rings; Supplementary Figure 1B) and between the activity evoked by radial ring and scene/place stimuli (Supplementary Figure 1C). We observed a different profile in the investigated regions (OPA-only, common and V3A-only). OPA-only showed a positive relationship between the activity evoked by radial rings and the activity evoked by both the flow field (*t*_27_ = 3.22; *p* = 0.003) and the scene/place stimulus (*t*_27_ = 3.65; *p* = 0.001). On the other hand, V3A-only and the common area showed significant positive correlations only in the radial ring vs. flow field comparison (V3A-only: *t*_27_ = 2.89; *p* = 0.007; common area: *t*_27_ = 4.32; *p* = 0.0002). Overall, while in all three regions we observed a positive relationship between the activity evoked by flow fields and the other two stimuli, OPA-only also showed a positive correlation between the activity evoked by radial ring and scene/place stimuli. These results could indicate that these regions are topographically organized according to a ventral-to-dorsal axis. In particular, the ventral-most region (OPA-only) might contain populations of nodes encoding both low- and high-level motion information together with scene-related information, and the dorsal-most regions (common area and pIPS/V3A) might contain neural populations mainly specialized in encoding low- and high-level motion information. Although the observed Pearson coefficients are positive and significant based on the *t*-tests, they are indicative of a quite low correlation (corresponding to a low amount of explained variance, ranking from 0.6 to 4.3%) so that caution is needed in the interpretation of the results (see “Discussion”).

Since we observed a low-level motion-selective response (during moving radial rings) in the probabilistically defined OPA and pIPS/V3A (see above), we wanted to control whether the individually defined areas OPA-only, common, and pIPS/V3A-only were equally motion sensitive. Plots in Fig. 7c show the bold response to the radial ring stimulus in the above-mentioned regions. We found that all these regions show a significant positive response (OPA-only: *t*_27_ = 6.58, *p* = 4.62 × 10^−7^; common: *t*_26_ = 3.94, *p* = 5.14 × 10^−4^; pIPS/V3A-only: *t*_27_ = 6.39, *p* = 7.54 × 10^−7^) with no difference among them (as highlighted by the absence of a main effect in the one-way ANOVA: *F* < 1; *p* > 0.8), indicating their undifferentiated involvement in processing visual motion.

## Discussion

We explored the relationship between scene/place perception and egomotion-compatible optic flow processing in a large sample of participants. We reanalyzed data from two well-known localizers, consisting in passive viewing of navigationally relevant stationary stimuli such as buildings and places (scene/place stimulus; Sulpizio et al. 2013, 2014, 2016a, 2018) and coherently moving fields of dots (high-level visual motion stimulus or flow fields, Pitzalis et al. 2010; Serra et al. 2019), previously used to localize scene- and egomotion-selective regions, respectively.

### Preference for navigational scenes in egomotion-selective areas V6+ and pIPS/V3A

One finding of the current study is that, among all the egomotion-selective areas, only pIPS/V3A and V6+ (although at different extent) showed a preference for navigational scenes compared to faces. However, whereas in pIPS/V3A we revealed the presence of a signal modulation as a function of the active blocks of the scene/place stimulus, the regional time course in V6+ revealed the presence of response deactivation in the face condition stronger than that observed in the scenes/place condition (Fig. 4). This might be explained by the extreme selectivity of the area for visual motion. In other words, static stimuli as faces could lead to the observed deactivations since they are completely irrelevant for the area. This is in line with the hypothesis that deactivation is the consequence of filtering out irrelevant information (Amedi et al. 2005). Under this hypothesis, perception of scenes/places might require much less filtering as compared to perception of faces, because environmental scenes, even if static, are often experienced during self-motion (navigation). On the other hand, contrary to scenes/places, it is unlikely that perception of faces can activate mental representation of self-motion. Thus, the observed deactivation may reflect different expectancies of self-motion evoked by static stimuli. Future studies will be needed to probe this hypothesis, for example, by testing the sensitivity of area V6 to different degrees of self-motion sensations not only during moving stimuli, but also during static images implying motion.

Beside the different functional profile shape observed here for V6+ and pIPS/V3A, in humans, these two areas have several properties suggesting a role in encoding navigationally relevant motion. The retinotopic area V6 (Galletti et al. 1999; Pitzalis et al. 2006) is more responsive to egomotion compatible than other types of coherent motion, with a preference for the translational egomotion (Cardin and Smith 2010; Pitzalis et al. 2010, 2013b), and it is strongly activated by the presence of stereoscopic depth cues associated with self-motion (Cardin and Smith 2011). The retinotopic area V3A (Tootell et al. 1997), which is strongly and directly connected with V6 in monkeys (Galletti et al. 2001) and humans (Tosoni et al. 2015; Serra et al. 2019), responds to changes of heading directions (Huang et al. 2012; Furlan et al. 2014) and, together with V6, is specialized in discounting extraretinal signals (coming from eye and head movements) from retinal visual motion in order to infer what is actually moving in the scene, in monkeys (Galletti et al. 1990; Galletti and Fattori 2003, 2018) and humans (Schindler and Bartels 2018a, b; Fischer et al. 2012; Nau et al. 2018). Interestingly, both V6 and V3A have been recently described in humans as involved in the “flow parsing mechanism” in a realistic virtual environment, being able to extract object motion information by subtracting out self-induced optical flow components (Pitzalis et al. 2020). In addition, the direct involvement of V6 and V3A in navigational tasks has been also suggested by anatomical (Kravitz et al. 2011) and resting-state functional connectivity data (Boccia et al. 2017a; Tosoni et al. 2015; Serra et al. 2019) and by functional connectivity analysis (Sherrill et al. 2015).

Taken together, present and previous evidence support the notion that motion processing is important not only on its own, but also in support to other higher-level functions such as self-motion perception and navigation. Our data extend this notion by demonstrating that the mere exposure to static, but navigationally relevant, scenes is able to trigger the activity of dorsal motion areas. Further work is required to address the precise types of scene information (i.e., tridimensional layout, geometry, field of view) that optimally drive these regions activity.

### Motion response in scene-selective areas

A second result of this study is that all the scene-selective areas PPA, RSC, and OPA show a significant response to coherently moving dots simulating self-motion as compared to patterns of randomly moving dots. Among these areas, however, only OPA exhibits a significant response to moving radial rings as compared to stationary rings, thus suggesting that PPA and RSC are not motion sensitive per se, but rather are specialized in processing visual motion produced by self-displacements. These results are compatible with prior studies that used static stimuli and suggested that PPA and RSC are modulated by the amount of experienced viewpoint change, being activated by the presentation of horizontally shifted scenes (Park and Chun 2009) and by imagined self-displacements to a new position (Sulpizio et al. 2013, 2016b).

On the other hand, the greater general involvement in motion processing observed in OPA is compatible with a previous study showing that this region represents motion information not only in scenes, but also during horizontal linear motion in phase-scrambled non-scene images (Korkmaz Hacialihafiz and Bartels 2015). Additionally, previous evidence suggested that OPA represents motion information relevant to visually guided navigation, such as first-person perspective motion, obstacle avoidance in the immediately visible scene (Kamps et al. 2016), and encoding of two essential kinds of information such as sense (left–right) and egocentric distance (proximal–distal) information (Dilks et al. 2011; Persichetti and Dilks 2016). More generally, the motion-related response here observed in OPA is compatible with its role in visually guided navigation (Persichetti and Dilks 2018). We argue that, while RSC and PPA may support scene recognition and spatial re-orientation by processing only high-level motion information, such as those related to egomotion, OPA may contribute to different stages of scene perception and thus may be involved in encoding any type of motion information (including local motion variations) necessary to guide navigation through the immediate environment.

### A common neural circuit for scene- and egomotion-related processing

The conjunction analysis between scene/place and flow field stimuli revealed the involvement of an occipito-parietal network that extended along the intraparietal sulcus and includes multiple atlas-based retinotopic maps such as the dorsal visual areas V7 (Tootell et al. 1998), V3B (Smith et al. 1998) and the lateral visual fields maps LO1 and LO2 (Larsson and Heeger 2006; Sayres and Grill-Spector 2008). Previous studies reported the involvement of these areas in several visuospatial processes. For example, V7 is modulated by spatial attention (Tootell et al. 1998) and, together with V3A, is sensitive to stereoscopic depth gradients (Cardin and Smith 2011). V3B is part of a large and functionally heterogeneous cortical territory hosting two well-known category-specific regions, such as the object-selective lateral occipital complex (LOC) (Malach et al. 1995; Grill-Spector et al. 1998; Levy et al. 2001; Hasson et al. 2003) and the kinetic occipital region (KO) (Dupont et al. 1997; Van Oostende et al. 1997). This portion of cortex, which also includes LO1 and LO2 (Larsson and Heeger 2006), has been previously described as implicated in the processing of kinetic boundaries (Dupont et al. 1997; Van Oostende et al. 1997; Larsson and Heeger 2006), boundaries defined by depth structure (Tyler et al. 2006), second-order pattern perception (Larsson et al. 2006), and visual shapes (Vinberg and Grill-Spector 2008). Note that previous studies reported that OPA was located in this cortical territory (Nasr et al. 2011; Huang and Sereno 2013; Silson et al. 2015; 2016). Taken together, previous and current results suggest that the dorsolateral parieto-occipital cortex plays a key role in extracting both complex visual patterns and high-order motion information.

Additionally, common activation for scene/place and flow field stimuli was observed in the ventromedial cortex, in correspondence and around the scene-selective areas PPA and RSC. Within the posterior parahippocampal cortex, two atlas-based retinotopic maps, PHC-1 and PHC-2 (Arcaro et al. 2009), which correspond to the areas VMV1-3 defined in the recent parcellation by Glasser et al. (2016), show a strong bias toward representations of peripheral eccentricities and are found to overlap with the functionally defined PPA (Arcaro et al. 2009). Furthermore, the scene-selective RSC includes, in its ventral-most portion (at the fundus of the calcarine sulcus), the area prostriata, which is preferentially activated by very fast motion particularly in the peripheral visual field (Mikellidou et al. 2017). All these pieces of evidence suggest that, beyond its general involvement in spatial processing, the ventromedial cortex may support motion detection through the peripheral visual field, which is an important prerequisite to estimate self-motion during spatial navigation.

### OPA and pIPS/V3A: distinct areas or a unique functional complex?

Our examination of the spatial relationship between OPA and pIPS/V3A revealed that pIPS/V3A extended more dorsally and medially with respect to OPA, although an overlapping area was found in most of the hemispheres. The degree of overlap varies across participants and hemispheres, with the peak of V3A falling within the boundaries of OPA in the majority of cases. Our comparison of scene- and egomotion-selectivity within these regions demonstrated a significant positive correlation suggesting the existence of a unique neural code in these regions, for both scene- and egomotion-relevant information. Also, the response to a low-level visual motion stimulus, indicating that both OPA and pIPS/V3A (and the overlapping area) responded stronger to moving than to static stimuli, confirms that these two regions have a similar functional profile.

Our data seem to suggest that OPA and pIPS/V3A play a unique role in representing both scene- and egomotion-relevant information. Recent evidence argues in favor of a unified framework in which several cognitive functions are supported by inter-connected neuronal networks, often involving the same neurons, whose activation changes dynamically according to the context (see Erlikhman et al. 2018 and Galletti and Fattori 2018 for recent reviews). This means that an area with a single functional property is an oversimplification. For example, object-related responses across the ventral and dorsal streams are likely to be similar in some circumstances, but show striking uniqueness and mutual independence in others, depending on the stimuli and task (Galletti and Fattori 2018). Accordingly, the current results seem to suggest the existence of a unique functional complex, including both OPA and pIPS/V3A, specialized in detecting moving items in the dynamic environment, likely with the aim of using egomotion-related visual cues to guide spatial navigation.

### Limitations of the study

There were a number of limitations in the present study which could be addressed in future work. First, we used the scenes/places > faces contrast to isolate scene-related activations, although the responses in this block-design contrast could be not limited to scene processing. Probably, a more specific contrast would have been scenes vs. scrambled images of these same scenes, in the hope that some of the low-level aspects of the scene images would be captured in the scrambled versions. However, some scene-selective regions, i.e., PPA and OPA, are also sensitive to low-level aspects of the scene images. In particular, a recent study (Watson et al. 2017) showed that intact and scrambled scenes produced highly similar patterns of response in OPA and PPA, thus indicating that visual properties play an important role in the topographic organization of these regions. Future studies are needed to understand how much the activity in the scene-selective regions is triggered by low-level features, which are essential for a quick discrimination of complex stimuli (Bacon-Macé et al. 2007; Greene and Oliva 2009) or by high-level category information which is important in supporting more abstract or specialized actions (e.g., navigation).

Another possible limitation concerns the correlation results. We observed a positive relationship between egomotion- and scene-related activity within the cortical territory hosting both pIPs/V3A and OPA, and a functional specialization within this portion of cortex as a function of the degree of interdependence with respect to a low-level motion activity, as indicated by significant *t*-tests. However, the low values of Pearson coefficients are indicative of a moderate correlation so that some caution is needed in concluding about the existence of a neural population encoding both motion- and scene-related activity.

Finally, we acknowledged that the lack of individual retinotopic mapping and the global nature of the analyses performed did not allow us to establish the exact spatial correspondence between the egomotion- and scene-selective regions and the retinotopically defined areas, so that our interpretations about differences between visual areas are speculative.

## Conclusions

World-centered motion is essential to keep track of changes in position and orientation while navigating in a complex and dynamic environment. Many years ago, it has been suggested (Ungerleider and Mishkin 1982; Goodale and Milner 1992) that dorsal and ventral streams are strictly segregated pathways, thought to be engaged in visual motion processing and scene recognition, respectively. However, here we failed to find a strict segregation of functions between the dorsal motion areas and the ventral scene-selective areas since they share a common neural substrate. Present findings point to the existence of an extended system supporting visually guided navigation, probably requiring the dynamic interaction between self-motion processing and perception of navigational scene. The general assumption behind this dynamic interaction is that motion information is a dominant cue for scene reconstruction and spatial updating (Britten 2008; Frenz et al. 2003; Medendorp et al. 2003). Consequently, the neural activity in scene-selective regions is modulated by visual motion cues, either when these are constituted by realistic environment (Korkmaz Hacialihafiz and Bartels 2015; Schindler and Bartels 2016; Pitzalis et al. 2020) or by coherently moving dot fields (present study). This points to a dynamic interplay between the dorsal and ventral visual pathways likely aimed at combining egomotion-related dynamic cues and scene-related static cues to coordinate visually guided navigation in real environments.

## Electronic supplementary material

Below is the link to the electronic supplementary material.

**Supplementary Figure** **1.** Node-to-node correlations. Node-to-node Pearson correlation between different stimuli, i.e., flow field and scene/place (**A**), flow field and radial ring (**B)** and radial ring and scene/place (**C**) within OPA-only, common and pIPS/V3A-only regions. Asterisks refer to *T* test versus zero performed after transforming Pearson correlation coefficients to z-values using the Fisher transform. *p < 0.001 (Bonferroni corrected). (TIFF 2175 kb)

**Supplementary Figure** **2.** Comparison of regional time courses. **A**. Across-scans and across-subjects average of activity of CSv (red line) and pPPA (blue line) is shown as a function of time (first 250 s) for both flow field (upper panel) and scene/place scans (lower panel). B. The same for pCi (red line) and RSC (blue line). (TIFF 5846 kb)

**Supplementary Figure** **3.** Results of the psychophysical validation. Plot shows the amount of self-motion sensation (SMS) and object-motion sensation (OMS) revealed by the ten-point Likert scale administered to a group of independent raters. Bars represent the median scores (± standard error) of SMS and OMS evoked by flow fields (coherent optic flow and random motion) and radial rings (coherent radial motion). **p < 10-8 (Bonferroni corrected); *p < 0.001 (Bonferroni corrected). (TIFF 1756 kb)

Supplementary material 4 (DOCX 17 kb)
