# Supplementary figures and images for: A common neural substrate for processing scenes and egomotion-compatible visual motion
Source: Brain Struct Funct. 2020 Jul 9;225(7):2091–110. doi: 10.1007/s00429-020-02112-8 (PMC7473967; doi:10.1007/s00429-020-02112-8)

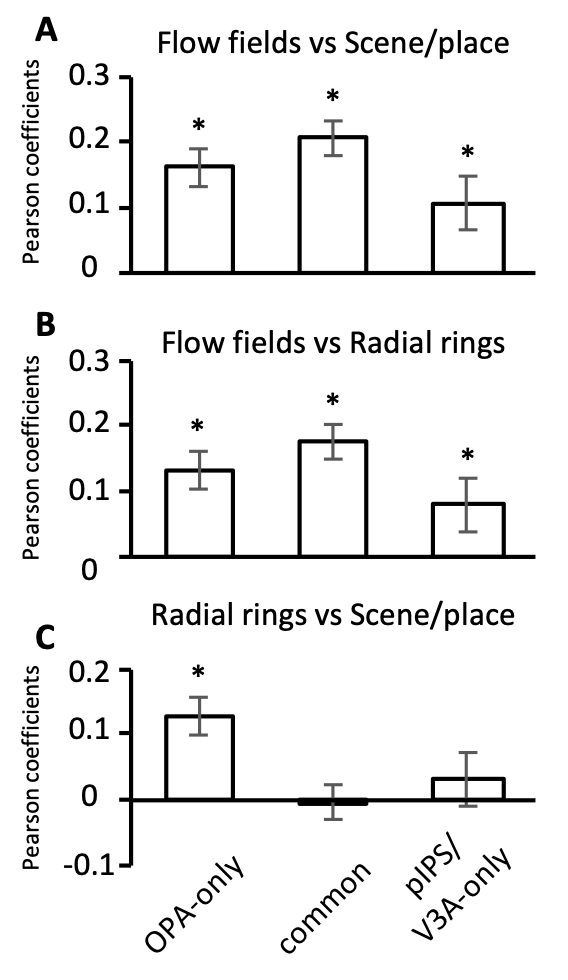

Supplement: Supplementary file 1 — Supplementary Figure 1. Node-to-node correlations. Node-to-node Pearson correlation between different stimuli, i.e., flow field and scene/place (A), flow field and radial ring (B) and radial ring and scene/place (C) within OPA-only, common and pIPS/V3A-only regions. Asterisks refer to T test versus zero performed after transforming Pearson correlation coefficients to z-values using the Fisher transform. *p < 0.001 (Bonferroni corrected). (TIFF 2175 kb) [file 429_2020_2112_MOESM1_ESM.tiff]

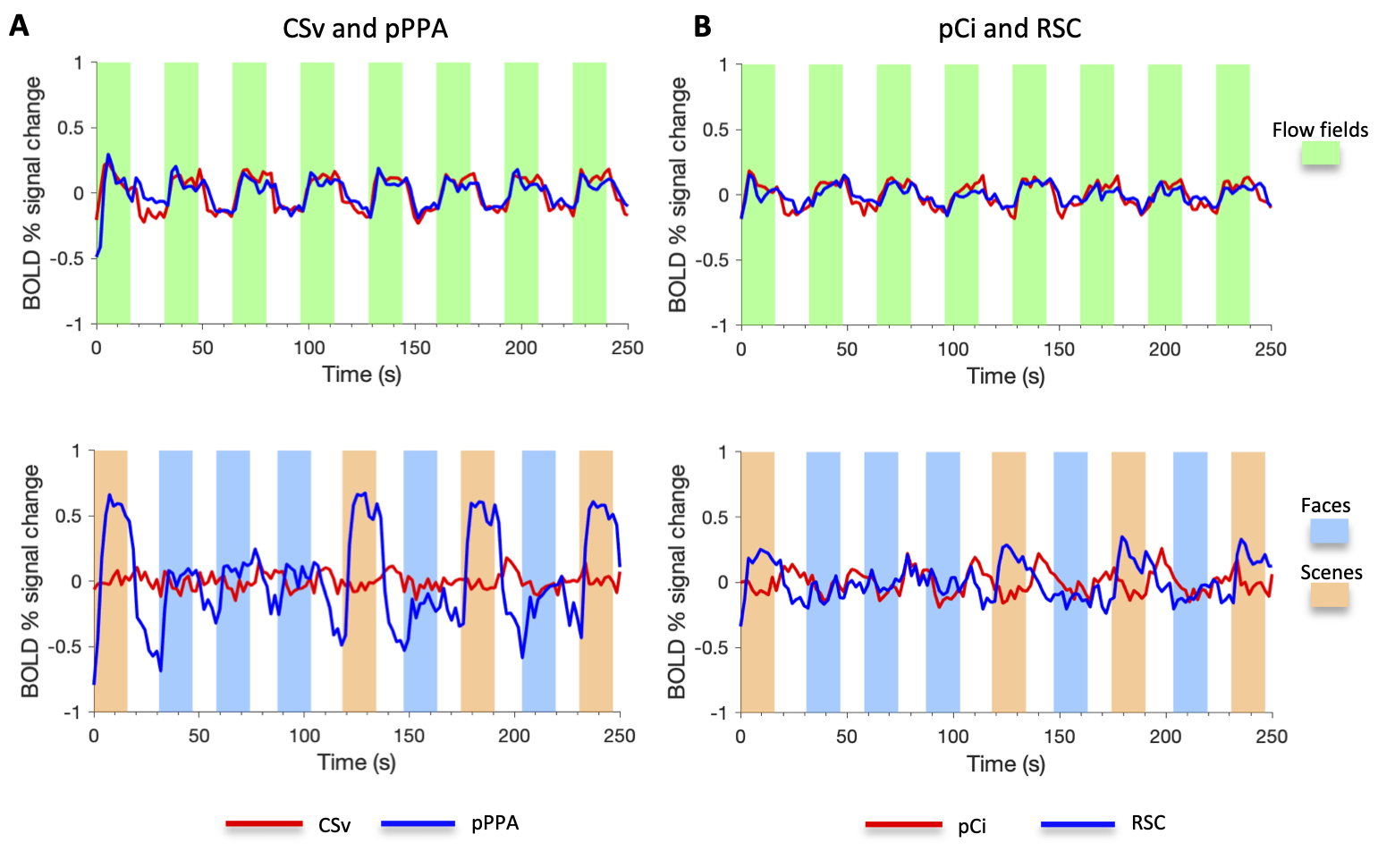

Supplement: Supplementary file 2 — Supplementary Figure 2. Comparison of regional time courses. A. Across-scans and across-subjects average of activity of CSv (red line) and pPPA (blue line) is shown as a function of time (first 250 s) for both flow field (upper panel) and scene/place scans (lower panel). B. The same for pCi (red line) and RSC (blue line). (TIFF 5846 kb) [file 429_2020_2112_MOESM2_ESM.tiff]

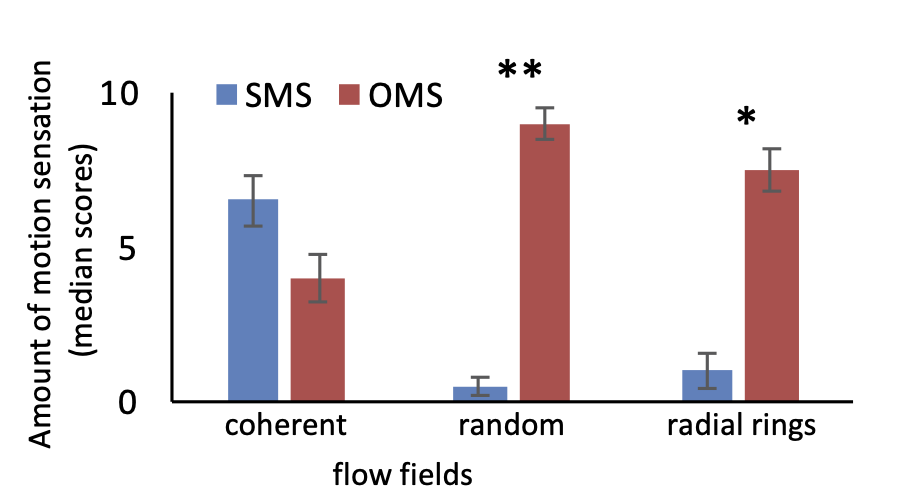

Supplement: Supplementary file 3 — Supplementary Figure 3. Results of the psychophysical validation. Plot shows the amount of self-motion sensation (SMS) and object-motion sensation (OMS) revealed by the ten-point Likert scale administered to a group of independent raters. Bars represent the median scores (± standard error) of SMS and OMS evoked by flow fields (coherent optic flow and random motion) and radial rings (coherent radial motion). **p < 10-8 (Bonferroni corrected); *p < 0.001 (Bonferroni corrected). (TIFF 1756 kb) [file 429_2020_2112_MOESM3_ESM.tiff]
