# Supplementary material for: A common neural substrate for processing scenes and egomotion-compatible visual motion
Source: Brain Struct Funct. 2020 Jul 9;225(7):2091–110. doi: 10.1007/s00429-020-02112-8 (PMC7473967; doi:10.1007/s00429-020-02112-8)
Supplement: Supplementary file 4 — Supplementary material 4 (DOCX 17 kb) [file 429_2020_2112_MOESM4_ESM.docx]

**A common neural substrate for processing scenes and egomotion-compatible visual motion**

Valentina Sulpizio, Gaspare Galati, Patrizia Fattori, Claudio Galletti, and Sabrina Pitzalis

**Supplementary Materials**

**Psychophysical Validation**

in this study, we have performed a psychophysical validation of the two visual motion stimuli (flow fields and radial rings, see Figure 1B-C) to verify and quantify the motion sensation experienced by our subjects. Due to the covid-19 pandemic, we had to opt for an online psychophysics. We administered a 3-item on-line questionnaire where we tested separately the ON and OFF phases of flow fields (i.e., coherent optic flow and random motion, respectively) and the ON phase of radial rings (i.e., radial motion). Specifically, we asked a group of independent raters (N = 14) to evaluate the intensity of self-motion sensation (SMS) and object-motion sensation (OMS) evoked by these three distinct motion stimuli through a ten-point Likert scale (no sensation – very high sensation). We then computed the medial value, so that scores lower than 5 indicated “low” sensation” and scores higher than 5 indicated “high” sensation.

**Results**

Supplementary Figure 3 shows the median scores across subjects, for SMS and OMS in flow fields (two conditions: coherent optic flow and random motion) and radial rings (one condition: radial motion). Note that the SMS and OMS scores are absent for the OFF phase of radial rings because this condition only includes static frames.

During coherent motion (ON phase in flow fields) participants judged higher SMS (median value = 6.5) as compared to OMS (median value = 4), although the difference between these scores were not significant (t _13_ = 1.26; p = 0.26). During random motion (OFF phase in flow fields) participants judged a pure OMS (median value = 9) being the SMS almost absent (median value = 0.5); the difference between OMS and SMS is highly significant (t _13_ = 14.02; p = 3.16 x 10^-9^). These results indicate that the coherent optic flow stimuli evoke both object and self-motion sensations. However, since the random motion evokes strong object motion but any self-motion sensation, our contrast coherent > random is in practice extremely effective in isolating the self-motion component. In support of this, we recently showed that at single-subject level, the flow field stimulus activated a network of six egomotion-selective areas (V6+, V3A, IPSmot/VIP, pCi, PIC, and CSv; see Serra et al. 2019) similar to that identified by Smith and coworkers using an egomotion-consistent/inconsistent stimulation (Wall et al. 2008; Cardin and Smith, 2010). Note that a strict comparison between the two visual stimuli cannot be done since there are no previous studies which have tested whether this egomotion-consistent/inconsistent stimulation induces self-motion and/or object motion as the flow field stimulus. Overall, combining all these observations, we think that the two optic flow stimulations, flow fields (Pitzalis et al., 2010; Pitzalis, Bozzacchi et al., 2013; Pitzalis, Fattori, & Galletti, 2013; Pitzalis, Sdoia, et al., 2013; Pitzalis, Sereno, et al., 2013), and egomotion-compatible/incompatible stimuli (Cardin & Smith, 2010; Wall & Smith, 2008), are comparable according to their sensitivity to map a network of egomotion-related areas.

During radial motion (ON phase in radial rings) participants judged SMS as very low (median value = 1) and OMS as very high (median value = 7.5). Paired t test revealed a significant difference between these scores (t 13 = 5.34; p = 1.33 x 10^-4^). We previously observed that this stimulus produced no illusory perception of self-motion (vection) (Pitzalis et al. 2010), as reported verbally by the subjects after each session. Present results confirmed that the quantify of self-motion sensation evoked by the radial ring stimulus is very low.

**Bibliography**

Cardin V, Smith AT (2010) Sensitivity of human visual and vestibular cortical regions to egomotion-compatible visual stimulation. Cerebral Cortex 20:1964–1973, http://doi.org/10.1093/cercor/bhp268.

Pitzalis S, Sereno MI, Committeri G, Fattori P, Galati G, Patria F, Galletti C (2010) Human v6: the medial motion area. Cereb Cortex 20:411–424. doi:10.1093/cercor/bhp112.

Pitzalis, S., Bozzacchi, C., Bultrini, A., Fattori, P., Galletti, C., & Di Russo, F. (2013). Parallel motion signals to the medial and lateral motion areas V6 and MT+. NeuroImage, 67, 89–100. http://doi.org/10.1016/j. neuroimage.2012.11.022

Pitzalis, S., Fattori, P., & Galletti, C. (2013). The functional role of the medial motion area V6. Frontiers in Behavioral Neuroscience, 6, 91. http://doi.org/10.3389/fnbeh.2012.00091

Pitzalis S, Sereno MI, Committeri G, Fattori P, Galati G, Tosoni A, Galletti, C (2013) The human homologue of macaque area V6A. Neuroimage 82:517–530.

Pitzalis S, Sdoia S, Bultrini A, Committeri G, Di Russo F, Fattori P, Galati G (2013) Selectivity to translational egomotion in human brain motion areas. PLoS One 8:1–14. http://doi.org/10.1371/journal. pone.0060241.

Serra C, Galletti C, Di Marco S, Fattori P, Galati G, Sulpizio V, Pitzalis S (2019) Egomotion-related visual areas respond to active leg movements. Hum Brain Mapp 40:3174–3191.

Wall, M. B., & Smith, A. T. (2008). The representation of egomotion in the human brain. Current Biology, 18(3), 191–194. http://doi.org/10.1016/ j.cub.2007.12.053
